# A case of trichodysplasia spinulosa related to ruxolitinib treated successfully with oral acitretin

**DOI:** 10.1002/ski2.276

**Published:** 2023-08-16

**Authors:** Priscilla M. Rosa‐Nieves, Robert Borucki, Corey Georgesen, Adam Sutton, Megan Arthur, Melodi Javid Whitley

**Affiliations:** ^1^ Department of Dermatology University of Nebraska Medical Center Omaha Nebraska USA; ^2^ School of Medicine Ponce Health Sciences University Ponce Puerto Rico USA; ^3^ Department of Dermatology Duke University School of Medicine Durham North Carolina USA

## Abstract

Trichodysplasia spinulosa (TS) is a rare disease that affects immunocompromised patients, characterized by hair‐like growths caused by *TS‐associated polyomavirus* infection. Little is known about specific immunosuppressive drugs that can precipitate the condition. We report a case of TS presenting after initiating the oral Janus‐associated kinase inhibitor (JAKi) ruxolitinib. A 67‐year‐old female with a history of allogeneic bone marrow transplant requiring immunosuppression with tacrolimus, prednisone and, more recently, ruxolitinib 5 mg twice daily due to Graft versus Host Disease presented to the clinic with a facial rash. The clinical and histopathological findings in the setting of immunosuppression were consistent with TS. Initial treatments were ineffective, but oral acitretin showed significant improvement after 3 months. Due to the close temporal relationship between the initiation of ruxolitinib and the development of TS, this case suggests that JAKis may contribute to TS development by suppressing the JAK‐signal transducer and activator of the transcription pathway's antiviral functions.

## Graphical abstract

Trichodysplasia spinulosa (TS) is a virally induced condition affecting immunocompromised individuals caused by TS‐associated polyomavirus. Patients present with perifollicular papules and keratin spicules that resemble tiny hairs and can progress to alopecic plaques. Given limited treatment options, our patient was started on oral acitretin and showed significant improvement.

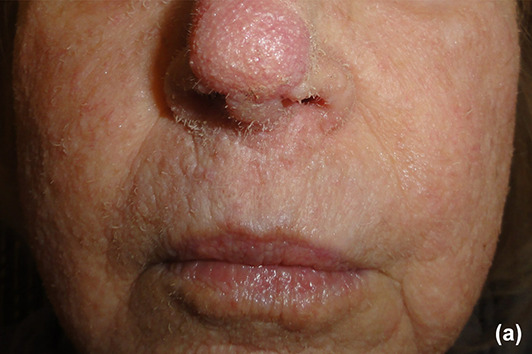

## 1 INTRODUCTION

Trichodysplasia spinulosa (TS) is a rare disease that affects immunocompromised patients, especially those with a history of solid organ transplantation, haematologic malignancy, and immunosuppression.^1^, ^2^ It is characterized by folliculocentric spinous processes and keratin spicules that resemble tiny hairs,^1^, ^2^, ^3^ due to infection with *TS‐associated polyomavirus*, an opportunistic infectious agent.^1^, ^2^ Although the exact pathophysiology of TS is not yet understood, the leading hypothesis involves virally‐induced hyperproliferation of inner root sheaths and the absence of dermal papillae, which are compatible with the follicular spines and alopecia seen in TS, respectively.^2^, ^3^ Many treatments have been deemed ineffective in the literature for treating TS, such as benzoyl peroxide, topical steroids, antibiotics, calcineurin inhibitors like topical tacrolimus, imiquimod, topical salicylic acid, ammonium lactate, topical urea, topical emollients, keratolytics, topical antifungals, and adapalene gel.^2^, ^3^ A few approaches have been successful: reducing immunosuppression, topical cidofovir 3%, oral valganciclovir, oral leflunomide, and oral acitretin.^1^, ^2^, ^4^ While TS is associated with immunosuppression, little is known about the role of individual drugs and onset of the disease. Here we report a case of TS presenting after initiating the oral Janus‐associated kinase inhibitor (JAKi) ruxolitinib.

## 2 CASE REPORT

**FIGURE 1:**
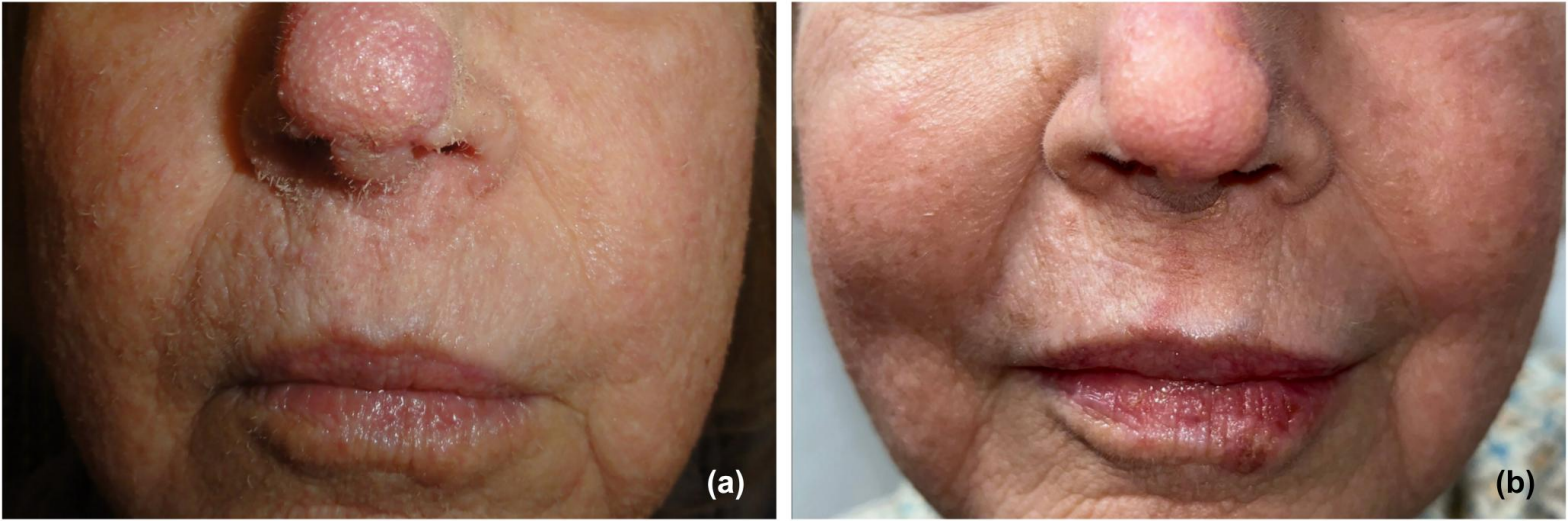
(a) Flesh‐colored papules and prominent keratinaceous follicular spines seen on the patient's cheeks and nose. (b) Significant improvement of TS after 3 months on oral acitretin 10 mg daily.

**FIGURE 2:**
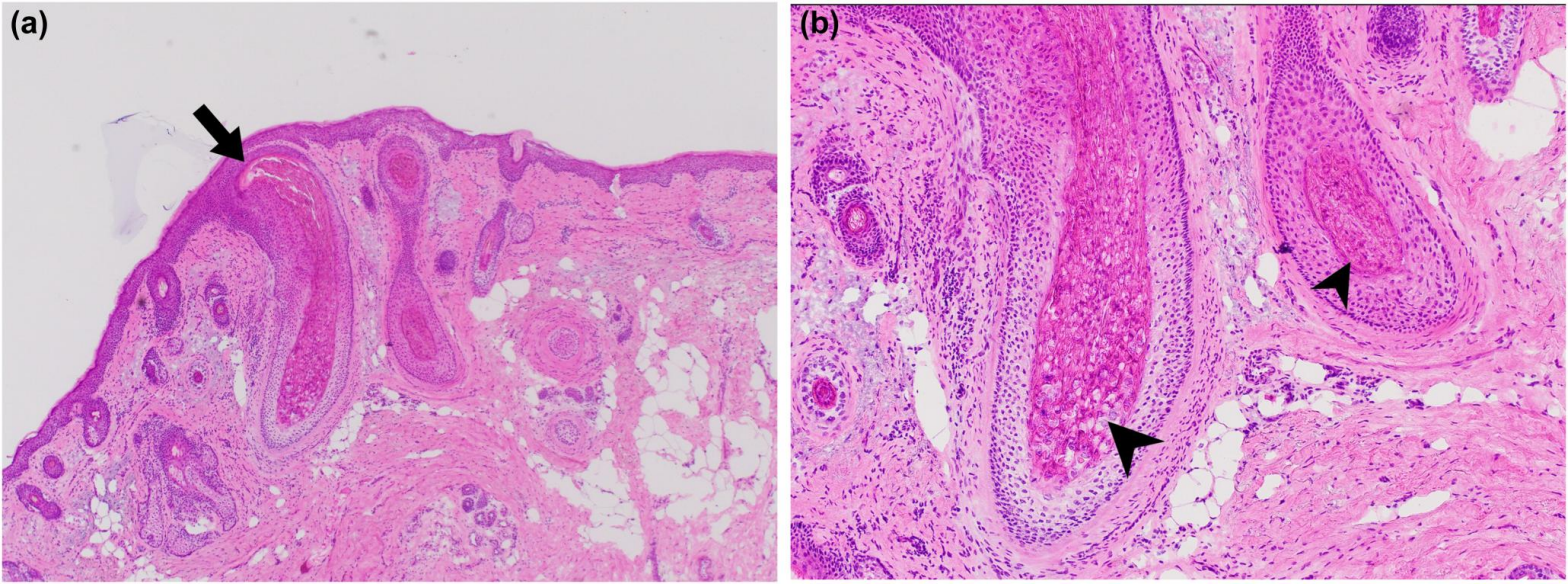
Eosinophilic trichohyalin granules and debris (arrowheads) within distended hair follicles (arrow) seen on a frozen section from Mohs Micrographic Surgery intraoperative slides; H&E, 4X (a) and 10X (b).

A 67‐year‐old female with a medical history of allogeneic bone marrow transplant (ABMT) from a related donor 18 months prior presented to the dermatology clinic with a facial rash. She reported a 2‐month history of skin‐colored bumps and rough skin on her face that felt like tiny hairs. On physical examination, there were flesh‐colored papules and prominent keratinaceous follicular spines on her forehead, cheeks, and nose (Figure 1a). Notably, the patient's post‐ABMT course was complicated by cutaneous and gastrointestinal chronic Graft versus Host Disease (GVHD), requiring immunosuppression with tacrolimus and prednisone. Due to refractory disease, she was initiated on ruxolitinib 5 mg twice daily 7 months prior to the current presentation to dermatology. While undergoing an unrelated Mohs Micrographic Surgery for an incidental basal cell carcinoma on her nasal dorsum, intraoperative frozen sections showed eosinophilic trichohyalin granules and debris within distended hair follicles (Figure 2). The clinical and histopathological findings in the setting of immunosuppression were consistent with TS. In this case, the reduction of immunosuppression was deemed inappropriate, given the presence of ongoing GVHD. Treatment was initiated with oral valganciclovir and tretinoin 0.05% cream. Due to a lack of improvement after 3 months, these agents were discontinued, and the patient was started on compounded topical cidofovir 3%. Unfortunately, this treatment was cost prohibitive and also proved unsuccessful. Ultimately, oral acitretin 10 mg daily was initiated. After 3 months, significant improvement was noted, and the patient remains improved on oral acitretin monotherapy (Figure 1b).

## 3 DISCUSSION

The JAK‐signal transducer and activator of transcription (JAK‐STAT) pathway provides innate and adaptive immunity against viral infections via cytokine secretion and controlled inflammation.^5^ Some viruses have JAK‐STAT escaping mechanisms that allow host infection.^5^ Ruxolitinib (Jakavi^®^ and Jakafi^®^) is an oral 1/2‐JAKi that suppresses the function of this pathway, thereby suppressing antiviral immunity.^6^ TS has previously been reported to occur following haematologic stem cell transplant.^4^ However, in this report, the patient had an onset of symptoms within 6 months of starting ruxolitinib, even though she had previously been on other immunosuppressive medications including tacrolimus and prednisone for over a year. This suggests that immunosuppression with JAKis specifically may lead to the development of TS. TS is caused by polyomavirus activation and therefore may emerge in the setting of JAK inhibition due to JAK‐STAT antiviral functions being suppressed, as evidenced in previous studies showing Human BK polyomavirus, Epstein Barr, and herpes simplex virus reactivation due to JAKis.^5^

We report a case of TS with a notable association with the initiation of the JAKi, ruxolitinib, the use of which is increasing in dermatology and beyond, treated successfully with oral acitretin, an effective new treatment modality for TS. As novel immunomodulatory medications come into broad clinical use, recognizing the breadth of cutaneous side effects is of importance to practicing dermatologists.

## CONFLICT OF INTEREST STATEMENT

The authors declare no conflicts of interest.

## AUTHOR CONTRIBUTIONS

**Priscilla M. Rosa‐Nieves**: Data curation (lead); writing—original draft (lead). **Robert Borucki**: Data curation (equal); writing—review and editing (equal). **Corey Georgesen**: Data curation (supporting); writing—review and editing (supporting). **Adam Sutton**: Data curation (equal); resources (equal); writing—review and editing (equal). **Megan Arthur**: Data curation (equal); writing—review and editing (equal). **Melodi Javid Whitley**: Conceptualization (lead); data curation (equal); supervision (lead); writing—review and editing (lead).

## ETHICS STATEMENT

Not applicable.

## Data Availability

Data sharing is not applicable to this article as no new data were created or analyzed in this study.
